# Mitochondrial and Metabolic Gene Expression in the Aged Rat Heart

**DOI:** 10.3389/fphys.2016.00352

**Published:** 2016-08-23

**Authors:** Gregory P. Barton, Joseph J. Sepe, Susan H. McKiernan, Judd M. Aiken, Gary M. Diffee

**Affiliations:** ^1^Balke Biodynamics Laboratory, Department of Kinesiology, University of Wisconsin-MadisonMadison, WI, USA; ^2^Departments of Agriculture, Food, and Nutritional Sciences, University of Alberta-EdmontonEdmonton, AB, Canada

**Keywords:** mitochondrial biogenesis, gene expression, exercise training, fatty acid metabolism, glucose metabolism

## Abstract

Aging is associated with a decline in cardiac function. Exercise intervention has been suggested as a way to improve this decrement. Age-related decline in cardiac function is associated with decreases in fatty acid oxidation, mitochondrial function, and AMP-activated protein kinase (AMPK) activity. The molecular mechanisms involved with age-related changes in mitochondrial function and substrate metabolism are poorly understood. We determined gene expression differences in hearts of Young (6 mo), Old (33 mo), and old exercise trained (Old + EXE) (34 mo) FBN rats, using Qiagen PCR arrays for Glucose, Fatty acid, and Mitochondrial metabolism. Old rats demonstrated decreased (*p* < 0.05) expression for key genes in fatty acid oxidation, mitochondrial function, and AMPK signaling. There were no differences in the expression of genes involved in glucose metabolism with age. These gene expression changes occurred prior to altered protein translation as we found no differences in the protein content of peroxisome proliferator activated receptor gamma, coactivators 1 alpha (PGC-1α), peroxisome proliferator activated receptor alpha (PPARα), and AMPKα_2_ between young and old hearts. Four months of exercise training did not attenuate the decline in the gene expression in aged hearts. Despite this lack of change in gene expression, exercise-trained rats demonstrated increased exercise capacity compared to their sedentary counterparts. Taken together, our results show that differential expression of genes associated with fatty acid metabolism, AMPK signaling and mitochondrial function decrease in the aging heart which may play a role in age-related declines in fatty acid oxidation, AMPK activity, and mitochondrial function in the heart.

## Introduction

Aging is associated with a decline in cardiac function in humans as well as rodents (Lakatta and Sollott, 2002). The decline in cardiac function with age is associated with changes in mitochondrial function and energy metabolism (Abu-Erreish et al., 1977; McMillin et al., 1993; Fannin et al., 1999; Kates et al., 2003; Bhashyam et al., 2007; Chakravarti et al., 2008). Since the myocardium derives nearly all of its energetic needs from the oxidation of pyruvate and fatty acids within the mitochondria, mitochondrial changes have great potential to contribute to cardiac dysfunction with age. Indeed, mitochondrial energetic deficiency with aging has been well-documented (McMillin et al., 1993; Fannin et al., 1999; Phaneuf and Leeuwenburgh, 2002; Chakravarti et al., 2008; Ventura-Clapier et al., 2008). The mechanisms of this mitochondrial dysfunction may include biogenesis that is inadequate to match the increasing demand (Goffart et al., 2004), as well as increased mitochondrial uncoupling and decreased substrate availability (Murray et al., 2004). Several studies have documented age-dependent impairment in the mitochondrial respiratory capacity (Kumaran et al., 2005; Navarro and Boveris, 2007).

Concomitant with mitochondria functional changes there is a change in cardiac substrate utilization during the aging process. At rest, fatty acids are the primary substrate for ATP supply in the myocardium and glucose uptake and oxidation supplies the remainder of the carbon substrates (Wisneski et al., 1985). There is some evidence that this substrate utilization changes with age (Abu-Erreish et al., 1977; McMillin et al., 1993; Sample et al., 2006), with most evidence indicating an age-related reduction in fatty acid oxidation, with the implication of increased reliance on glucose, although this is not universally seen (Sample et al., 2006). Interestingly, it has been determined that glucose utilization itself does not increase (Abu-Erreish et al., 1977; McMillin et al., 1993; Kates et al., 2003).

The molecular mechanisms underlying age-related changes in mitochondrial function or substrate energy metabolism is largely unknown. Decreases in mRNA levels of peroxisome proliferator-activated receptor (PPARα), and some of its downstream targets (i.e., CPT-1, β-hydroxyacyl dehydrogenase) have been observed with age (Iemitsu et al., 2002). In addition, peroxisome proliferator activated receptor γ coactivator-1α (PGC-1α) protein content is reduced in the aging left ventricle (Turdi et al., 2010). PPARα is a transcription factor for genes involved with fatty acid transport and β-oxidation (Huss and Kelly, 2004). PGC-1α, a co-transcription factor is known to stimulate mitochondrial DNA replication and the coding for genes involved with oxidative phosphorylation (Huss and Kelly, 2004). Another mechanism for age-related changes in substrate metabolism may be related to AMP-activated protein kinase (AMPK) activity. Dually activated by AMP and by upstream phosphorylation, this kinase promotes fatty acid oxidation, glucose uptake, and glycogenolysis while it inhibits anabolic processes such as fatty acid synthesis (Munday et al., 1988; Coven et al., 2003; Hawley et al., 2003, 2005; Scott et al., 2004). There is evidence that AMPK activity declines with age (Gonzalez et al., 2004; Turdi et al., 2010). Changes in the expression, protein content, or activity of PPARα, PGC-1α, and AMPK can help explain some of the known changes in substrate metabolism and mitochondrial function with age. However, these processes are the result of complex pathways that require the coordinated expression and function of a large number of genes and proteins, but there are few studies that have examined the effects of aging on the expression of the comprehensive array of genes that are associated with substrate metabolism and mitochondrial function.

Exercise training is known to improve indices of cardiac function in humans (Fortney et al., 1992; Seals et al., 1994) and rodents (Spurgeon et al., 1983; Iemitsu et al., 2002). Specifically, these functional improvements include, increased contractile function (Spurgeon et al., 1983; Fortney et al., 1992; Seals et al., 1994; Iemitsu et al., 2002) and increased maximal oxygen uptake (VO_2max_) (Ogawa et al., 1992; Seals et al., 1994; Stratton et al., 1994). There is some evidence that exercise can alter the metabolic phenotype of the aging heart. Exercise training in aging Wistar rats increased AMPK activity, PPARα mRNA and protein content, and proteins involved in fatty acid oxidation in the young and aged heart (Iemitsu et al., 2002; Rimbaud et al., 2009; Dobrzyn et al., 2013). Also, lifelong voluntary wheel running in mice increased electron transport chain (ETC) complex IV gene expression (Bronikowski et al., 2003). There is molecular evidence that exercise training can increase the mRNA and protein levels of important proteins involved with fatty acid oxidation and oxidative phosphorylation, these markers suggest that these metabolic pathways may be improved with exercise training (Iemitsu et al., 2002; Bronikowski et al., 2003; Rimbaud et al., 2009; Dobrzyn et al., 2013). However, substrate metabolism and mitochondrial oxidative phosphorylation in the heart are regulated by the transcription, translation, and activity of many genes in order to optimally function.

Therefore, the aims of this study was to: (1) determine the effects of age on the expression of a large number of genes related to the pathways of glucose and fatty acid metabolism, and mitochondrial function; and (2) determine whether exercise training could mitigate age-related changes in the expression of metabolic and mitochondrial genes in the aging rat heart. We hypothesize that expression of genes associated with the pathways of fatty acid metabolism, AMPK signaling, and mitochondrial function will decrease with age and that the addition of exercise training in these aged rats will mitigate this decrease in gene expression.

## Materials and methods

### Animals

Male Fischer 344 x Brown Norway hybrid rats (FBN), were obtained from the National Institute on Aging colony at Harlan Industries (Indianapolis, IN). The FBN hybrid rat is a long-lived strain with a median life-span of 33 months and a maximum life-span of 40 months. The FBN rat is considered a “healthy aging model” widely used and highly recommended for gerontological research. All rats were confined to standard size rodent cages and housed 2 rats per cage. Rats had access to food and water *ad libitum* and were acclimated to reverse daylight (12 h dark, 12 h light). Body weights and average food intake were monitored through the course of the study. Rats were randomly assigned to one of three groups: Young (6 month), Old (33–34 month), and Old + Exercise (Old+EXE) (33–34 month). Animal housing and handling was carried out under the guidelines of the University of Wisconsin-Madison Institutional Animal Care and Use Committees and conducted in pathogen-free facilities that are accredited by the American Association of Accreditation of Laboratory Animal Care.

### Tissue collection

Old and Old + EXE hearts were removed and flash frozen in liquid nitrogen 72 h from the last maximal exercise test in order to control for transient gene expression changes due to acute exercise (Neufer and Dohm, 1993; Pilegaard et al., 2000). From the Old (*n* = 9) and Old + EXE (*n* = 9) rats, 5 hearts were randomly selected for qRT-PCR, Western blot, and citrate synthase experiments, while Young (*n* = 5) hearts were used for molecular analysis.

### Maximal exercise testing

Assessment of peak exercise capacity occurred each month beginning at 30 months until 34 months of age. The exercise test started with a treadmill speed at 5 m/min and progressively increased by 3 m/min at each exercise stage. Animals ran at each new treadmill speed for 3 min to assure steady-state values. To encourage the rats to run, the treadmill was equipped with an electric shock grid at the rear of the treadmill. The shock grid was set to deliver a 0.2 mA current, which gives an uncomfortable shock but does not physically harm or injure the rat. The test was terminated when animals were no longer able to maintain position on the treadmill, and the highest speed was recorded as peak exercise capacity. Blood lactate levels were measured during the graded exercise tests. Blood lactate levels were used to quantify relative exercise intensity. At the end of each 3 min interval, animals were briefly removed from the treadmill, immobilized with the tail extended, the lateral tail vein pierced, and a drop (~25 μL) of blood was collected on a lactate strip inserted into a lactate meter (Lactate Plus Meter, Nova Biomedical). Blood lactate values were recorded at each exercise intensity level during the exercise test.

### Training protocol

Trained animals were exercised on a motor driven treadmill during their 12 h dark cycle. Before training began, animals were treadmill acclimatized for 2 weeks at 5 m/min for 5 min during the first week and at 5 m/min for 10 min the second week. After acclimatization, maximal exercise tests were performed and blood lactate measurements were taken at each successive speed on the treadmill during the test. The endurance trained rats began exercise training at 30 months of age with a training program that following 2 weeks of treadmill acclimation consisted of 30 min/day, 5 days per week at a speed that corresponded to each animal's lactate threshold. Thus, the training speed was adjusted each month based on the results of the maximal exercise tests (above). All exercise training sessions included a 3 min warm-up period at 5 m/min. No negative stimuli (electric shock) were used during the daily exercise training of the animals to minimize stress involved in exercise for these aging animals.

### RNA isolation and cDNA preparation

Rats were terminated using isoflurane/pneumothorax euthanasia and hearts were removed and flash frozen in liquid nitrogen. Total RNA from each rat was isolated from the left ventricular free wall using RNeasy® Microarray Tissue Mini Kit (Qiagen) according to the manufacturer instruction. Immediately after elution RNA concentration and purity was measured spectrophotometrically (Beckman-Coulter). For each sample 700 ng of total RNA was reversed transcribed using the RT^2^ First Strand Kit (Qiagen). The reaction was performed at 42°C for 15 min followed by a termination step at 95°C for 5 min. cDNA was stored at −20°C until qRT-PCR.

### qRT-PCR data analysis

A total of 251 genes of interest across three PCR arrays from Qiagen were assayed for this study (Glucose Metabolism RT^2^ Profiler PCR Array, Fatty Acid Metabolism RT^2^ Profiler PCR Array and RT^2^ Custom PCR Array for mitochondrial function). A list of all of the genes contained in each array can be found in Tables 1–3. PCR was performed on Step-One Plus PCR system (Applied Biosystems), according to the manufacturer's instructions. For data analysis, the ΔΔCt method was used with the aid of a Microsoft Excel spreadsheet containing algorithms and a student's *t*-test was used to analyze differences in fold-changes in gene expression provided by the manufacturer (Hassmann-Poznańska et al., 2013; Wu et al., 2013; Okada et al., 2014). The ΔΔCt method for calculating differences in gene expression are as follows: (ΔCt = Ct^GOI^ − Ct^AVG HKG^, where GOI is the gene of interest and HKG is the housekeeping gene selected. ΔΔCt = (ΔCt (Experimental group) −ΔCt (Control)). The housekeeping gene selected for data analysis was Ribosomal protein, large P1 (Rplp1). Fold-changes were then calculated and expressed as log-normalized ratios of values from Old/Young, Old + EXE/Young and Old + EXE/Old heart tissues.

**Table 1:** **Gene list Glucose metabolism array**.

**Symbol**	**Description**
Acly	ATP citrate lyase
Aco1	Aconitase 1, soluble
Aco2	Aconitase 2, mitochondrial
Agl	Amylo-1,6-glucosidase, 4-alpha-glucanotransferase
Aldoa	Aldolase A, fructose-bisphosphate
Aldob	Aldolase B, fructose-bisphosphate
Aldoc	Aldolase C, fructose-bisphosphate
Bpgm	2,3-bisphosphoglycerate mutase
Cs	Citrate synthase
Dlat	Dihydrolipoamide S-acetyltransferase
Dld	Dihydrolipoamide dehydrogenase
Dlst	Dihydrolipoamide S-succinyltransferase (E2 component of 2-oxo-glutarate complex)
Eno1	Enolase 1, (alpha)
Eno2	Enolase 2, gamma, neuronal
Eno3	Enolase 3, beta, muscle
Fbp1	Fructose-1,6-bisphosphatase 1
Fbp2	Fructose-1,6-bisphosphatase 2
Fh	Fumarate hydratase 1
G6pc	Glucose-6-phosphatase, catalytic subunit
G6pc3	Glucose 6 phosphatase, catalytic, 3
G6pd	Glucose-6-phosphate dehydrogenase
Galm	Galactose mutarotase (aldose 1-epimerase)
Gapdh	Glyceraldehyde-3-phosphate dehydrogenase
Gapdhs	Glyceraldehyde-3-phosphate dehydrogenase, spermatogenic
Gck	Glucokinase
Gpi	Glucose phosphate isomerase
Gsk3a	Glycogen synthase kinase 3 alpha
Gsk3b	Glycogen synthase kinase 3 beta
Gys1	Glycogen synthase 1, muscle
Gys2	Glycogen synthase 2
H6pd	Hexose-6-phosphate dehydrogenase (glucose 1-dehydrogenase)
Hk2	Hexokinase 2
Hk3	Hexokinase 3 (white cell)
Idh1	Isocitrate dehydrogenase 1 (NADP+), soluble
Idh2	Isocitrate dehydrogenase 2 (NADP+), mitochondrial
Idh3a	Isocitrate dehydrogenase 3 (NAD+) alpha
Idh3b	Isocitrate dehydrogenase 3 (NAD+) beta
Idh3g	Isocitrate dehydrogenase 3 (NAD), gamma
Mdh1	Malate dehydrogenase 1, NAD (soluble)
Mdh1b	Malate dehydrogenase 1B, NAD (soluble)
Mdh2	Malate dehydrogenase 2, NAD (mitochondrial)
Ogdhl	Oxoglutarate dehydrogenase-like
Pc	Pyruvate carboxylase
Pck1	Phosphoenolpyruvate carboxykinase 1 (soluble)
Pck2	Phosphoenolpyruvate carboxykinase 2 (mitochondrial)
Pdha2	Pyruvate dehydrogenase (lipoamide) alpha 2
Pdhb	Pyruvate dehydrogenase (lipoamide) beta
Pdhx	Pyruvate dehydrogenase complex, component X
Pdk1	Pyruvate dehydrogenase kinase, isozyme 1
Pdk2	Pyruvate dehydrogenase kinase, isozyme 2
Pdk3	Pyruvate dehydrogenase kinase, isozyme 3
Pdk4	Pyruvate dehydrogenase kinase, isozyme 4
Pdp2	Pyruvate dehyrogenase phosphatase catalytic subunit 2
Pdpr	Pyruvate dehydrogenase phosphatase regulatory subunit
Pfkl	Phosphofructokinase, liver
Pgam2	Phosphoglycerate mutase 2 (muscle)
Pgk1	Phosphoglycerate kinase 1
Pgk2	Phosphoglycerate kinase 2
Pgls	6-phosphogluconolactonase
Pgm1	Phosphoglucomutase 1
Pgm2	Phosphoglucomutase 2
Pgm3	Phosphoglucomutase 3
Phka1	Phosphorylase kinase, alpha 1
Phkb	Phosphorylase kinase, beta
Phkg1	Phosphorylase kinase, gamma 1
Phkg2	Phosphorylase kinase, gamma 2 (testis)
Pklr	Pyruvate kinase, liver, and RBC
Prps1	Phosphoribosyl pyrophosphate synthetase 1
Prps1l1	Phosphoribosyl pyrophosphate synthetase 1-like 1
Pygl	Phosphorylase, glycogen, liver
Pygm	Phosphorylase, glycogen, muscle
Rbks	Ribokinase
Rpia	Ribose 5-phosphate isomerase A
Sdha	Succinate dehydrogenase complex, subunit A, flavoprotein (Fp)
Sdhb	Succinate dehydrogenase complex, subunit B, iron sulfur (Ip)
Sdhc	Succinate dehydrogenase complex, subunit C, integral membrane protein
Sdhd	Succinate dehydrogenase complex, subunit D, integral membrane protein
Sucla2	Succinate-CoA ligase, ADP-forming, beta subunit
Suclg1	Succinate-CoA ligase, alpha subunit
Suclg2	Succinate-CoA ligase, GDP-forming, beta subunit
Taldo1	Transaldolase 1
Tkt	Transketolase
Tpi1	Triosephosphate isomerase 1
Ugp2	UDP-glucose pyrophosphorylase 2

**Table 2:** **Gene list for Fatty Acid metabolism array**.

**Symbol**	**Description**
Acaa1a	Acetyl-Coenzyme A acyltransferase 1A
Acaa2	Acetyl-Coenzyme A acyltransferase 2
Acad10	Acyl-Coenzyme A dehydrogenase family, member 10
Acad11	Acyl-Coenzyme A dehydrogenase family, member 11
Acad9	Acyl-Coenzyme A dehydrogenase family, member 9
Acadl	Acyl-Coenzyme A dehydrogenase, long-chain
Acadm	Acyl-Coenzyme A dehydrogenase, C-4 to C-12 straight chain
Acads	Acyl-Coenzyme A dehydrogenase, C-2 to C-3 short chain
Acadsb	Acyl-Coenzyme A dehydrogenase, short/branched chain
Acadvl	Acyl-Coenzyme A dehydrogenase, very long chain
Acat1	Acetyl-coenzyme A acetyltransferase 1
Acat2	Acetyl-Coenzyme A acetyltransferase 3
Acot12	Acyl-CoA thioesterase 12
Acot2	Acyl-CoA thioesterase 2
Acot3	Acyl-CoA thioesterase 3
Acot7	Acyl-CoA thioesterase 7
Acot8	Acyl-CoA thioesterase 8
Acot9	Acyl-CoA thioesterase 9
Acox1	Acyl-Coenzyme A oxidase 1, palmitoyl
Acox2	Acyl-Coenzyme A oxidase 2, branched chain
Acox3	Acyl-Coenzyme A oxidase 3, pristanoyl
Acsbg1	Acyl-CoA synthetase bubblegum family member 1
Acsbg2	Acyl-CoA synthetase bubblegum family member 2
Acsl1	Acyl-CoA synthetase long-chain family member 1
Acsl3	Acyl-CoA synthetase long-chain family member 3
Acsl4	Acyl-CoA synthetase long-chain family member 4
Acsl5	Acyl-CoA synthetase long-chain family member 5
Acsl6	Acyl-CoA synthetase long-chain family member 6
Acsm2a	Acyl-CoA synthetase medium-chain family member 2
Acsm3	Acyl-CoA synthetase medium-chain family member 3
Acsm4	Acyl-CoA synthetase medium-chain family member 4
Acsm5	Acyl-CoA synthetase medium-chain family member 5
Aldh2	Aldehyde dehydrogenase 2 family (mitochondrial)
Bdh1	3-hydroxybutyrate dehydrogenase, type 1
Bdh2	3-hydroxybutyrate dehydrogenase, type 2
Cpt1a	Carnitine palmitoyltransferase 1a, liver
Cpt1b	Carnitine palmitoyltransferase 1b, muscle
Cpt1c	Carnitine palmitoyltransferase 1c
Cpt2	Carnitine palmitoyltransferase 2
Crat	Carnitine acetyltransferase
Crot	Carnitine O-octanoyltransferase
Decr1	2,4-dienoyl CoA reductase 1, mitochondrial
Decr2	2,4-dienoyl CoA reductase 2, peroxisomal
Echs1	Enoyl Coenzyme A hydratase, short chain, 1, mitochondrial
Eci2	Enoyl-Coenzyme A delta isomerase 2
Ehhadh	Enoyl-Coenzyme A, hydratase/3-hydroxyacyl Coenzyme A dehydrogenase
Fabp1	Fatty acid binding protein 1, liver
Fabp2	Fatty acid binding protein 2, intestinal
Fabp3	Fatty acid binding protein 3, muscle, and heart
Fabp4	Fatty acid binding protein 4, adipocyte
Fabp5	Fatty acid binding protein 5, epidermal
Fabp6	Fatty acid binding protein 6, ileal
Fabp7	Fatty acid binding protein 7, brain
Gcdh	Glutaryl-Coenzyme A dehydrogenase
Gk	Glycerol kinase
Gk2	Glycerol kinase 2
Gpd1	Glycerol-3-phosphate dehydrogenase 1 (soluble)
Gpd2	Glycerol-3-phosphate dehydrogenase 2, mitochondrial
Hadha	Hydroxyacyl-Coenzyme A dehydrogenase^a^
Hmgcl	3-hydroxymethyl-3-methylglutaryl-Coenzyme A lyase
Hmgcs1	3-hydroxy-3-methylglutaryl-Coenzyme A synthase 1 (soluble)
Hmgcs2	3-hydroxy-3-methylglutaryl-Coenzyme A synthase 2 (mitochondrial)
Lipe	Lipase, hormone sensitive
Lpl	Lipoprotein lipase
Mcee	Methylmalonyl CoA epimerase
Mut	Methylmalonyl-Coenzyme A mutase
Oxct2a	3-oxoacid CoA transferase 2A
Pecr	Peroxisomal trans-2-enoyl-CoA reductase
Ppa1	Pyrophosphatase (inorganic) 1
Prkaa1	Protein kinase, AMP-activated, alpha 1 catalytic subunit
Prkaa2	Protein kinase, AMP-activated, alpha 2 catalytic subunit
Prkab1	Protein kinase, AMP-activated, beta 1 non-catalytic subunit
Prkab2	Protein kinase, AMP-activated, beta 2 non-catalytic subunit
Prkaca	Protein kinase, cAMP-dependent, catalytic, alpha
Prkacb	Protein kinase, cAMP dependent, catalytic, beta
Prkag1	Protein kinase, AMP-activated, gamma 1 non-catalytic subunit
Prkag2	Protein kinase, AMP-activated, gamma 2 non-catalytic subunit
Prkag3	Protein kinase, AMP-activated, gamma 3 non-catalytic subunit
Slc27a1	Solute carrier family 27 (fatty acid transporter), member 1
Slc27a2	Solute carrier family 27 (fatty acid transporter), member 2
Slc27a3	Solute carrier family 27 (fatty acid transporter), member 3
Slc27a4	Solute carrier family 27 (fatty acid transporter), member 4
Slc27a5	Solute carrier family 27 (fatty acid transporter), member 5
Slc27a6	Solute carrier family 27 (fatty acid transporter), member 6

a Hydroxyacyl-Coenzyme A dehydrogenase 3-ketoacyl-Coenzyme A thiolase/enoyl-Coenzyme A hydratase (trifunctional protein), alpha subunit.

**Table 3:** **Gene list for the Custom array**.

**Symbol**	**Description**
Ppargc1	Peroxisome proliferator-activated receptor gamma, coactivator 1 alpha
Nrf1	Nuclear respiratory factor 1
Tfam	Transcription factor A, mitochondrial
Sir2	Sirtuin (Silent mating type information regulation 2 homolog) 2
Esrra	Estrogen related receptor, alpha
Prkaa2	Protein kinase, AMP-activated, alpha 2 catalytic subunit
CREB1	cAMP response element binding protein 1
CaMK4	Calcium/calmodulin-dependent protein kinase IV
SOD2	Superoxide dismutase 2, mitochondrial
UCP2	Uncoupling protein 2 (mitochondrial proton carrier)
UCP3	Uncoupling protein 3 (mitochondrial proton carrier)
Bax	Bcl2–associated X protein
Bcl2	B-cell CLL/lymphoma 2
Casp3	Caspase 3
Gabpa	GA binding protein transcription factor (alpha subunit) (i.e., NRF-2a)
UCP1	Uncoupling protein 1 (mitochondrial proton carrier)
Ppara	Peroxisome proliferator activated receptor alpha
Slc2a4	Solute carrier family 2 (facilitated glucose transporter) member 4
CD36	CD 36 molecule (thrombospondin receptor)
Acacb	Acetyl-Coenzyme A carboxylase beta
Mlycd	Malonyl-CoA decarboxylase
Ndufa1	NADH dehydrogenase (ubiquinone) 1 alpha subcomplex, 1
Ndufa10	NADH dehydrogenase (ubiquinone) 1 alpha subcomplex, 10
Ndufa11	NADH dehydrogenase (ubiquinone) 1 alpha subcomplex, 11
Ndufa2	NADH dehydrogenase (ubiquinone) 1 alpha subcomplex, 2
Ndufa5	NADH dehydrogenase (ubiquinone) 1 alpha subcomplex, 5
Ndufa6	NADH dehydrogenase (ubiquinone) 1 alpha subcomplex, 6
Ndufa7	NADH dehydrogenase (ubiquinone) 1 alpha subcomplex, 7
Ndufa8	NADH dehydrogenase (ubiquinone) 1 alpha subcomplex, 8
Ndufa9	NADH dehydrogenase (ubiquinone) 1 alpha subcomplex, 9
Ndufab1	NADH dehydrogenase (ubiquinone) 1 alpha/beta subcomplex, 1
Ndufb2	NADH dehydrogenase (ubiquinone) 1 beta subcomplex, 2
Ndufb3	NADH dehydrogenase (ubiquinone) 1 beta subcomplex, 3
Ndufb5	NADH dehydrogenase (ubiquinone) 1 beta subcomplex, 5
Ndufb6	NADH dehydrogenase (ubiquinone) 1 beta subcomplex, 6
Ndufb7	NADH dehydrogenase (ubiquinone) 1 beta subcomplex, 7
Ndufb8	NADH dehydrogenase (ubiquinone) 1 beta subcomplex, 8
Ndufb9	NADH dehydrogenase (ubiquinone) 1 beta subcomplex, 9
Ndufc2	NADH dehydrogenase (ubiquinone) 1 subcomplex unknown, 2
Ndufs1	NADH dehyrogenase (ubiquinone) Fe-S protein 1
Ndufs2	NADH dehyrogenase (ubiquinone) Fe-S protein 2
Ndufs3	NADH dehyrogenase (ubiquinone) Fe-S protein 3
Ndufs4	NADH dehyrogenase (ubiquinone) Fe-S protein 4
Ndufs6	NADH dehyrogenase (ubiquinone) Fe-S protein 6
Ndufs7	NADH dehyrogenase (ubiquinone) Fe-S protein 7
Ndufs8	NADH dehyrogenase (ubiquinone) Fe-S protein 8
Ndufv1	NADH dehydrogenase (ubiquinone) flavoprotein 1
Ndufv2	NADH dehyrogenase (ubiquinone) flavoprotein 2
Bcs1l	BCS1-like yeast
Cyc1	Cytochrome c-1
Uqcrb	Ubiquinol-cytochrome c reductase binding protein
Uqcrc1	Ubiquinol-cytochrome c reductase core protein 1
Uqcrc2	Ubiquinol-cytochrome c reductase core protein 2
Uqcrfs1	Ubiquinol-cytochrome c reductase, Rieske iron-sulfur polypeptide 1
Uqcrh	Ubiquinol-cytochrome c reductase hinge protein
Uqcrq	Ubiquinol-cytochrome c reductase, complex III subunit VII
Cox15	COX 15 homolog, cytochrome c oxidase assembly protein (yeast)
Cox17	COX 17 cytochrome c oxidase assembly homolog (*S. cerevisiae*)
Cox4i1	Cytochrome c oxidase subunit IV isoform 1
Cox4i2	Cytochrome c oxidase subunit IV isoform 2
Cox5a	Cytochrome c oxidase subunit Va
Cox5b	Cytochrome c oxidase subunit Vb
Cox6a1	Cytochrome c oxidase subunit VIa polypeptide 1
Cox6a2	Cytochrome c oxidase subunit VIa polypeptide 2
Cox6c	Cytochrome c oxidase subunit VIc
Cox7a2	Cytochrome c oxidase subunit VIIa polypeptide 2
Cox7a2l	Cytochrome c oxidase subunit VIIa polypeptide 2 like
Cox7b	Cytochrome c oxidase subunit VIIb polypeptide
Cox8a	Cytochrome c oxidase subunit VIIIa
Cox8c	Cytochrome c oxidase subunit VIIIc
mfn1	Mitofusin 1
mfn2	Mitofusin 2
fis1	Fission 1 (mitochondrial outer membrane) homolog (*S. cerevisiae*)
lonp1	Lon protease
Aifm2	Apoptosis-inducing factor, mitochondrial-associated 2
Bcl2l1	Bcl2-like 1
Clpb	ClpB caseinolytic peptidase B homolog (*E. coli*) (i.e. HSP 78)
pnpt1	Polyribonucleotide nucleotidyltransferase 1
Me1	Malic enzyme 1, NADP(+)-dependent, cytosolic
Foxo3	Forkhead box O3
Camkk2	Calcium/calmodulin-dependent protein kinase kinase 2,beta
Stk11	Serine/threonine kinase 11 (i.e., LKB1)
Ppargc1b	Peroxisome proliferator-activated receptor gamma, coactivator 1 beta
Tp53	Tumor protein p53

### Western blotting

Whole cell lysate from isolated left ventricle was prepared using CelLytic™ MT Cell Lysis Reagent and 1:100 dilution of Protease Inhibitor Cocktail (Sigma, St. Louis, MO). The protein concentration was determined using Bio-Rad Protein Assay (BioRad, Hercules, CA). Thirty micrograms of whole cellular protein per lane was separated by SDS-PAGE with a 4–12% Bis-Tris Criterion™ XT gel (XT MOPS running buffer) and blotted onto a nitrocellulose membrane. The membrane was incubated with blocking buffer (5% non-fat dry milk/TBS/0.1% Tween 20) at room temperature for 1 h. The membrane was then probed with the primary antibodies diluted in blocking buffer overnight at 4°C. Subsequently, membranes were incubated with horseradish peroxidase-conjugated anti-rabbit antibody diluted 1:2000 in blocking buffer. Blots were developed with Clarity™ Western ECL substrate (BioRad, Hercules, CA) and imaged with GE ImageQuant LAS-4000 (GE Healthcare Bio-Sciences, Pittsburgh, PA). The image of the blots was uploaded and densitometry analysis was done with Image Studio Lite (LI-COR Biosciences, Lincoln, NE). Protein content was measured from the densitometry units from PGC-1α, AMPKα_2_,PPARα, and normalized to vinculin.

### Antibodies

Rabbit anti-PGC-1α, AMPKα_2_, and Vinculin antibodies were acquired from Cell Signaling Technology, Inc., Beverly, MA. Rabbit anti-PPARα antibody was purchased from Santa Cruz Biotechnology, Inc., Santa Cruz, CA.

### Citrate synthase activity

Maximal citrate synthase (CS) activity was determined in left ventricular homogenates using (Citrate Synthase Assay Kit, Sigma-Aldrich, St Louis, MO, USA) according to the manufacturer's protocol with absorbance kinetically measured at 412 nm at baseline and after addition of oxaloacetate (Sigma-Aldrich). CS activity was normalized to protein content with tissue protein determined using the Bio-Rad protein assay.

### Statistical analysis

A two-tailed Student's *t*-test was used for analyzing differences in gene expression and exercise performance. A one-way ANOVA was utilized to analyze differences in protein content and citrate synthase activity. *Post-hoc* analysis was performed when the one-way ANOVA was significant using Tukey's LSD to assess between group differences. Significance was determined at *p* < 0.05.

## Results

### Effects of exercise training on exercise performance

**Figure 1:**
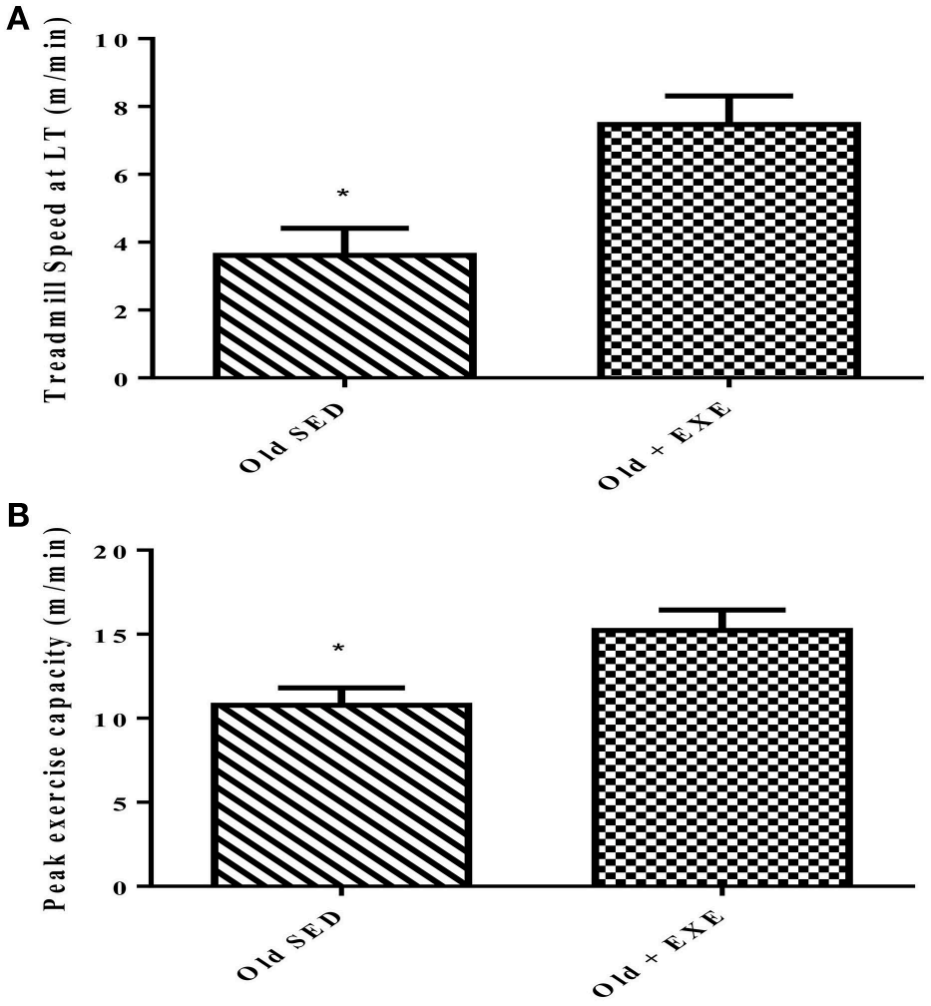
**Effects of exercise training on peak exercise capacity in 34 month old FBN rats**. Values are the Means ± S.E.M of treadmill speed and peak exercise capacity of trained and sedentary rats. **(A)** We measured exercise capacity as a function of treadmill speed at lactate threshold (LT) **(B)** Peak exercise capacity was measured as the maximal speed at the end of the maximal exercise testing. Old SED (*n* = 9), Old + EXE (*n* = 9). ^*^P < 0.05.

The effect of 4 months of exercise training on exercise performance in 34 month old trained (Old + EXE) FBN rats compared to sedentary (Old SED) rats was significant (Figure 1). We measured exercise capacity in two different ways; (1) treadmill speed at lactate threshold (LT) and (2) peak exercise capacity (the final treadmill speed achieved during the maximal exercise test). Exercise training significantly improved lactate threshold (*p* < 0.01) in the Old + EXE rats compared to Old SED rats (Figure 1A). Peak exercise capacity was also greater (*p* = 0.01) in the Old + EXE rats compared to Old SED rats (Figure 1B).

### Gene expression changes with age

**Figure 2:**
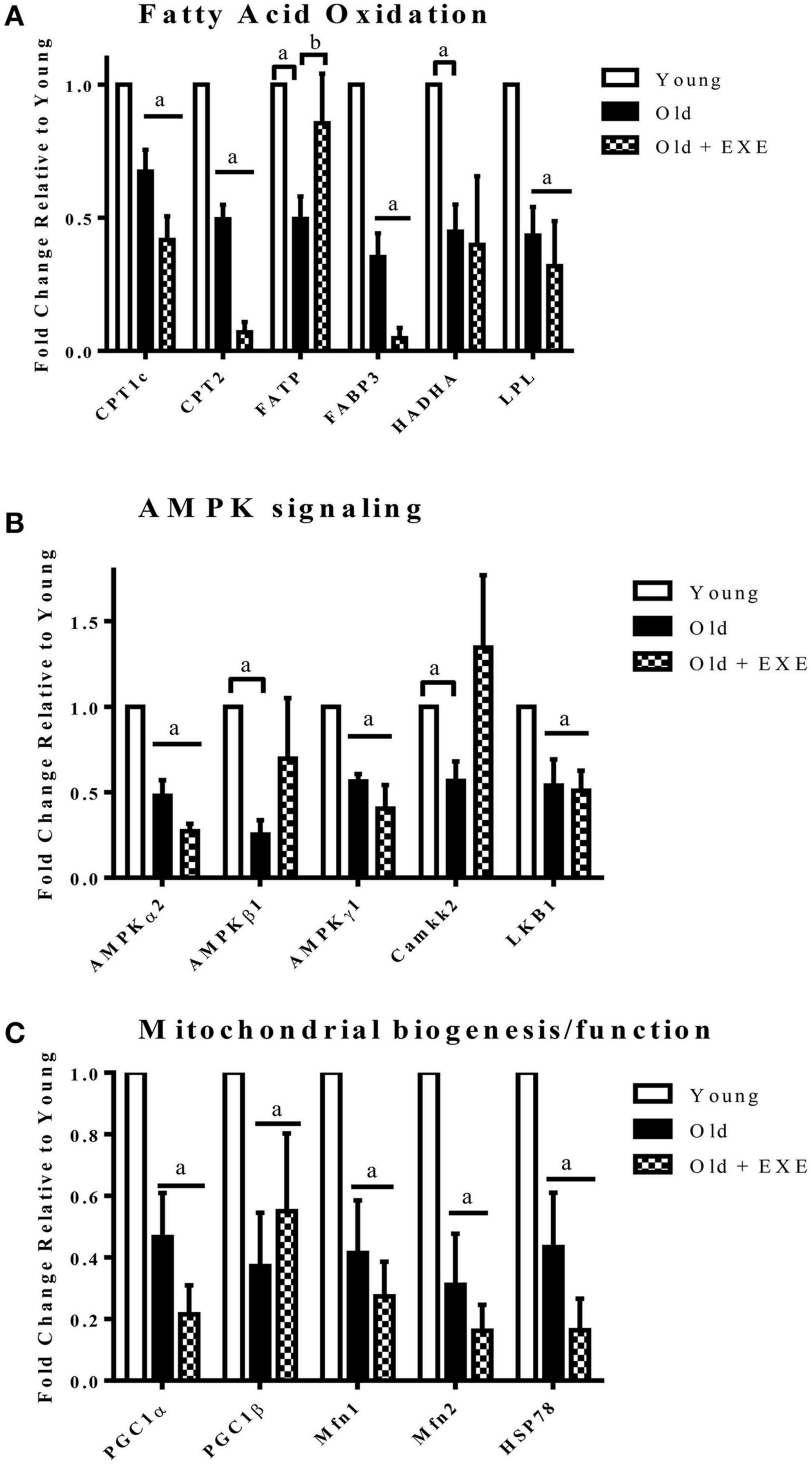
**Fold changes in gene expression Old and Old + EXE hearts relative to young hearts (Values are the Means ±S.E.M, *n* = 5 per group). (A)** Genes involved in fatty acid transport and oxidation decline with age and this attenuation is not mitigated with exercise. (CPT1c, Carnitine-palmitoyl transferase 1c; CPT2, Carnitine palmitoyl transferase 2; FATP, Fatty acid transporter; FABP3, Fatty acid binding protein 3; HADHA, Hydroxyacyl-Coenzyme A dehydrogenase/3-ketoacyl-Coenzyme A thiolase/enoyl-CoA hydratase (trifunctional protein); LPL, Lipoprotein lipase). **(B)** AMPK signaling decrements with age involve changes in the gene expression of AMPK subunits and upstream kinases. (AMPKα2, Protein kinase; AMP, activated alpha 2 catalytic subunit; AMPKβ1, Protein kinase; AMP, activated beta 1 non-catalytic subunit; AMPKγ1, Protein kinase; AMP, activated gamma 1 non-catalytic sununit; Camkk2, Calcium/calmodulin-dependent protein kinase kinase 2 beta; LKB1, Liver kinase B 1). **(C)** Mitochondrial biogenesis and maintenance of mitochondrial function decline with age and exercise does not appear to improve the attenuation. (PGC1α, Peroxisome proliferator—activated receptor gamma, coactivator 1 alpha; PGC1β, Peroxisome proliferator—activator receptor gamma, coactivator 1 beta; Mfn1, Mitofusin 1; Mfn2, Mitofusin 2; HSP78, Heat shock protein 78). ^a^*P* < 0.05 vs. Young, ^b^*P* < 0.05 vs. Old + EXE.

To determine the effects of age on the expression of genes involved in cardiac glucose and fatty acid metabolism as well as mitochondrial function we performed qRT-PCR using three different PCR arrays. Table 4 shows all genes in the energetic pathways for which expression was significantly different between Old and Young rats. Between the three arrays there were a total of 44 genes that were differentially expressed (*p* < 0.05) in Old compared to Young. Of the 44 genes differentially expressed, 42 of these genes had decreased expression in Old compared to Young. Notably, the expression profile of three important pathways of energy production were altered with age; fatty acid oxidation (FAO), mitochondrial biogenesis, and AMPK signaling. There were no significant changes in glucose metabolism gene expression with age. Figure 2 shows changes in expression with age in key genes involved in the pathways of FAO, mitochondrial biogenesis, and AMPK signaling.

**Table 4:** **Age-related gene expression changes in the left ventricle**.

**Gene name**	**Gene symbol**	**Fold Regulation**	***p*-value**
**FA METABOLISM**			
Acyl-CoA thioesterase 12	Acot12	2.88	0.026
Acyl-CoA thioesterase 7	Acot7	–1.86	0.017
Acyl-CoA thioesterase 8	Acot8	–1.44	0.02
Acyl-Coenzyme A oxidase 1, palmitoyl	Acox1	–2.1	0.032
Acyl-CoA synthetase bubblegum family member 1	Acsbg1	2.09	0.034
Acyl-CoA synthetase long-chain family member 6	Acsl6	–1.98	0.029
3-hydroxybutyrate dehydrogenase, type 1	Bdh1	–2.55	0.036
Carnitine palmitoyltransferase 1c	Cpt1c	–1.49	0.027
Carnitine palmitoyltransferase 2	Cpt2	–2.02	0.001
Carnitine acetyltransferase	Crat	–2.69	0.011
2,4-dienoyl CoA reductase 1, mitochondrial	Decr1	–2.05	0.03
Enoyl Coenzyme A hydratase, short chain, 1, mitochondrial	Echs1	–2.39	0.004
Enoyl-Coenzyme A delta isomerase 2	Eci2	–1.79	0.049
Enoyl-Coenzyme A, hydratase/3-hydroxyacyl Coenzyme A dehydrogenase	Ehhadh	–3.29	0.034
Fatty acid binding protein 3, muscle and heart	Fabp3	–2.84	0.017
Glutaryl-Coenzyme A dehydrogenase	Gcdh	–1.97	0.013
Glycerol-3-phosphate dehydrogenase 2, mitochondrial	Gpd2	–2.64	0.028
Hydroxyacyl-Coenzyme A dehydrogenase/3-ketoacyl-Coenzyme A thiolase/enoyl-Coenzyme A hydratase (trifunctional protein), alpha subunit	Hadha	–2.23	0.035
3-hydroxymethyl-3-methylglutaryl-Coenzyme A lyase	Hmgcl	–1.51	0.03
Lipase, hormone sensitive	Lipe	–2.14	0.03
Lipoprotein lipase	Lpl	–2.31	0.025
Peroxisomal trans-2-enoyl-CoA reductase	Pecr	–1.67	0.013
Pyrophosphatase (inorganic) 1	Ppa1	–1.78	0.03
Protein kinase, AMP-activated, alpha 2 catalytic subunit	Prkaa2	–2.09	0.034
Protein kinase, AMP-activated, beta 1 non-catalytic subunit	Prkab1	–3.96	0.041
Protein kinase, AMP-activated, beta 2 non-catalytic subunit	Prkab2	–1.7	0.041
Protein kinase, cAMP-dependent, catalytic, alpha	Prkaca	–2.03	0.005
Protein kinase, cAMP dependent, catalytic, beta	Prkacb	–1.65	0.031
Protein kinase, AMP-activated, gamma 1 non-catalytic subunit	Prkag1	–1.77	0.002
Solute carrier family 27 (fatty acid transporter), member 1	Slc27a1	–2.02	0.03
**MITOCHONDRIAL FUNCTION**			
Peroxisome proliferator—activated receptor gamma, coactivator 1 alpha	Ppargc1	–2.14	0.03
NADH dehydrogenase (ubiquinone) Fe-S protein 7	Ndufs7	–2.24	0.015
Ubiquinol - cytochrome c reductase, complex III subunit VII	Uqcrq	–1.48	0.05
COX17 cytochrome c oxidase assembly homolog (*S. cerevisiae*)	Cox17	–1.68	0.011
Cytochrome c oxidase subunit IV isoform 1	Cox4i1	–1.72	0.02
Cytochrome c oxidase subunit VIIa polypeptide 2 like	Cox7a2l	–1.35	0.049
Mitofusin 1	mfn1	–2.41	0.01
Mitofusin 2	mfn2	–3.21	0.028
Clpb caseinolytic peptidase B homolog (*E. coli*) (i.e. HSP78)	clpb	–2.3	0.039
Calcium/calmodulin-dependent protein kinase kinase 2, beta	Camkk2	–1.77	0.017
Serine/threonine kinase 11 (i.e. LKB1)	Stk11	–1.86	0.029
Peroxisome proliferator - activated receptor gamma, coactivator 1 beta	Ppargc1b	–2.69	0.012
Tumor protein p53	Tp53	–2.19	0.025

### Effects of exercise training on gene expression in aged hearts

Differences in gene expression were observed between Old and Old + EXE rat hearts (Table 5). 70 genes associated with glucose metabolism, FA metabolism and mitochondrial function were altered with exercise training in cardiac tissue of old rats compared to that of old sedentary rats. Of these 70 genes, only three were upregulated (*Prkaa1, Slc27a1, Slc27a4*), while 67 genes were downregulated with exercise training and involved genes from the Kreb's cycle, glucose transport (Glut4), fatty acid oxidation, and the mitochondrial electron transport chain.

**Table 5:** **Exercise-related changes in gene expression in Old FBN rats**.

**Gene name**	**Gene symbol**	**Fold Regulation**	***p*-value**
**GLUCOSE METABOLISM**			
Amylo-1,6-glucosidase, 4-alpha-glucanotransferase	Agl	–6.15	0.024
Aldolase A, fructose-bisphosphate	Aldoa	–5.28	0.036
Enolase 1, (alpha)	Eno1	–4.1	0.042
Enolase 2, gamma, neuronal	Eno2	–3.27	0.019
Glucose phosphate isomerase	Gpi	–5.59	0.005
Glycogen synthase kinase 3 alpha	Gsk3a	–3.17	0.046
Isocitrate dehydrogenase 3 (NAD+) beta	Idh3b	–7.7	0.04
Isocitrate dehydrogenase 3 (NAD), gamma	Idh3g	–5.41	0.027
Malate dehydrogenase 2, NAD (mitochondrial)	Mdh2	–6.31	0.009
Pyruvate dehydrogenase kinase, isozyme 1	Pdk1	–9.29	0.004
Pyruvate dehydrogenase kinase, isozyme 2	Pdk2	–6.48	0.006
Phosphoglucomutase 3	Pgm3	–1.7	0.037
Phosphorylase kinase, gamma 1	Phkg1	–2.78	0.011
Ribose 5-phosphate isomerase A	Rpia	–2.38	0.018
Succinate dehydrogenase complex, subunit A, flavoprotein (Fp)	Sdha	–3.93	0.038
Succinate-CoA ligase, ADP-forming, beta subunit	Sucla2	–5.64	0.021
Solute carrier family 2 (facilitated glucose transporter), member 4	Slc2a4	–10.45	0.025
**FA METABOLISM**			
Acetyl-Coenzyme A acyltransferase 1A	Acaa1a	–9.79	0.001
Acetyl-Coenzyme A acyltransferase 2	Acaa2	–11.7	0.029
Acyl-Coenzyme A dehydrogenase family, member 10	Acad10	–7.02	0.002
Acyl-Coenzyme A dehydrogenase family, member 11	Acad11	–14.38	0.002
Acyl-Coenzyme A dehydrogenase family, member 9	Acad9	–8.81	0.008
Acyl-Coenzyme A dehydrogenase, long-chain	Acadl	–21.18	0.002
Acyl-Coenzyme A dehydrogenase, C-4 to C-12 straight chain	Acadm	–15.17	0.009
Acyl-Coenzyme A dehydrogenase, C-2 to C-3 short chain	Acads	–17.09	0.001
Acyl-Coenzyme A dehydrogenase, short/branched chain	Acadsb	–7.75	0.007
Acyl-Coenzyme A dehydrogenase, very long chain	Acadvl	–9.49	0.002
Acetyl-coenzyme A acetyltransferase 1	Acat1	–14.79	0.004
Acetyl-Coenzyme A acetyltransferase 3	Acat2	–6.25	0.003
Acyl-CoA thioesterase 2	Acot2	–18.92	< 0.001
Acyl-CoA thioesterase 3	Acot3	–3.52	0.024
Acyl-CoA thioesterase 7	Acot7	–4.43	0.027
Acyl-CoA thioesterase 8	Acot8	–2.68	0.002
Acyl-CoA thioesterase 9	Acot9	–5.41	0.001
Acyl-Coenzyme A oxidase 1, palmitoyl	Acox1	–5.16	0.007
Acyl-Coenzyme A oxidase 3, pristanoyl	Acox3	–4.47	0.015
Acyl-CoA synthetase long-chain family member 1	Acsl1	–18.06	< 0.001
Acyl-CoA synthetase long-chain family member 3	Acsl3	–3.6	0.008
Acyl-CoA synthetase long-chain family member 4	Acsl4	–2.46	0.011
Acyl-CoA synthetase long-chain family member 5	Acsl5	–2.23	0.001
Acyl-CoA synthetase long-chain family member 6	Acsl6	–5.54	0.01
3-hydroxybutyrate dehydrogenase, type 1	Bdh1	–10.48	0.04
3-hydroxybutyrate dehydrogenase, type 2	Bdh2	–5.03	0.008
Carnitine palmitoyltransferase 1a, liver	Cpt1a	–3.04	0.033
Carnitine palmitoyltransferase 1b, muscle	Cpt1b	–7.93	0.003
Carnitine palmitoyltransferase 2	Cpt2	–7.02	0.004
Carnitine acetyltransferase	Crat	–4.27	0.018
Fatty acid binding protein 3, muscle and heart	Fabp3	–7.31	0.034
Protein kinase, AMP-activated, alpha 1 catalytic subunit	Prkaa1	1.83	0.042
Protein kinase, cAMP-dependent, catalytic, alpha	Prkaca	–2.89	0.041
Protein kinase, cAMP dependent, catalytic, beta	Prkacb	–1.43	0.047
Solute carrier family 27 (fatty acid transporter), member 1	Slc27a1	1.73	0.033
Solute carrier family 27 (fatty acid transporter), member 4	Slc27a4	3.15	0.002
CD36 molecule (thrombospondin receptor)	CD36	–2.97	0.001
Peroxisome proliferator activated receptor alpha	Ppara	–9.28	0.029
**MITOCHONDRIAL FUNCTION**			
Sirtuin (silent mating type information regulation 2 homolog) 1 (*S. cerevisiae*)	Sirt1	–3.27	0.036
NADH dehyrogenase (ubiquinone) 1 alpha subcomplex, 1	Ndufa1	–2.24	0.04
NADH dehyrogenase (ubiquinone) 1 alpha subcomplex, 6 (B14)	Ndufa6	–4.7	0.041
NADH dehyrogenase (ubiquinone) 1 alpha subcomplex, 7	Ndufa7	–5.67	0.033
NADH dehyrogenase (ubiquinone) 1 alpha subcomplex, 8	Ndufa8	–10.41	0.005
NADH dehyrogenase (ubiquinone) 1 alpha subcomplex, 9	Ndufa9	–5.71	0.018
NADH dehyrogenase (ubiquinone) 1, alpha/beta subcomplex, 1	Ndufab1	–5.67	0.001
NADH dehyrogenase (ubiquinone) 1 beta subcomplex, 2	Ndufb2	–4.49	0.006
NADH dehyrogenase (ubiquinone) 1 beta subcomplex, 5	Ndufb5	–4.72	0.032
NADH dehyrogenase (ubiquinone) 1 beta subcomplex, 8	Ndufb8	–4.92	0.01
NADH dehyrogenase (ubiquinone) Fe-S protein 6	Ndufs6	–2.94	0.047
Cytochrome c-1	Cyc1	–5.47	0.033
Ubiquinol-cytochrome c reductase core protein 2	Uqcrc2	–5.38	0.016
Ubiquinol-cytochrome c reductase, Rieske iron-sulfur polypeptide 1	Uqcrfs1	–5.11	0.045

### Western blot analysis

**Figure 3:**
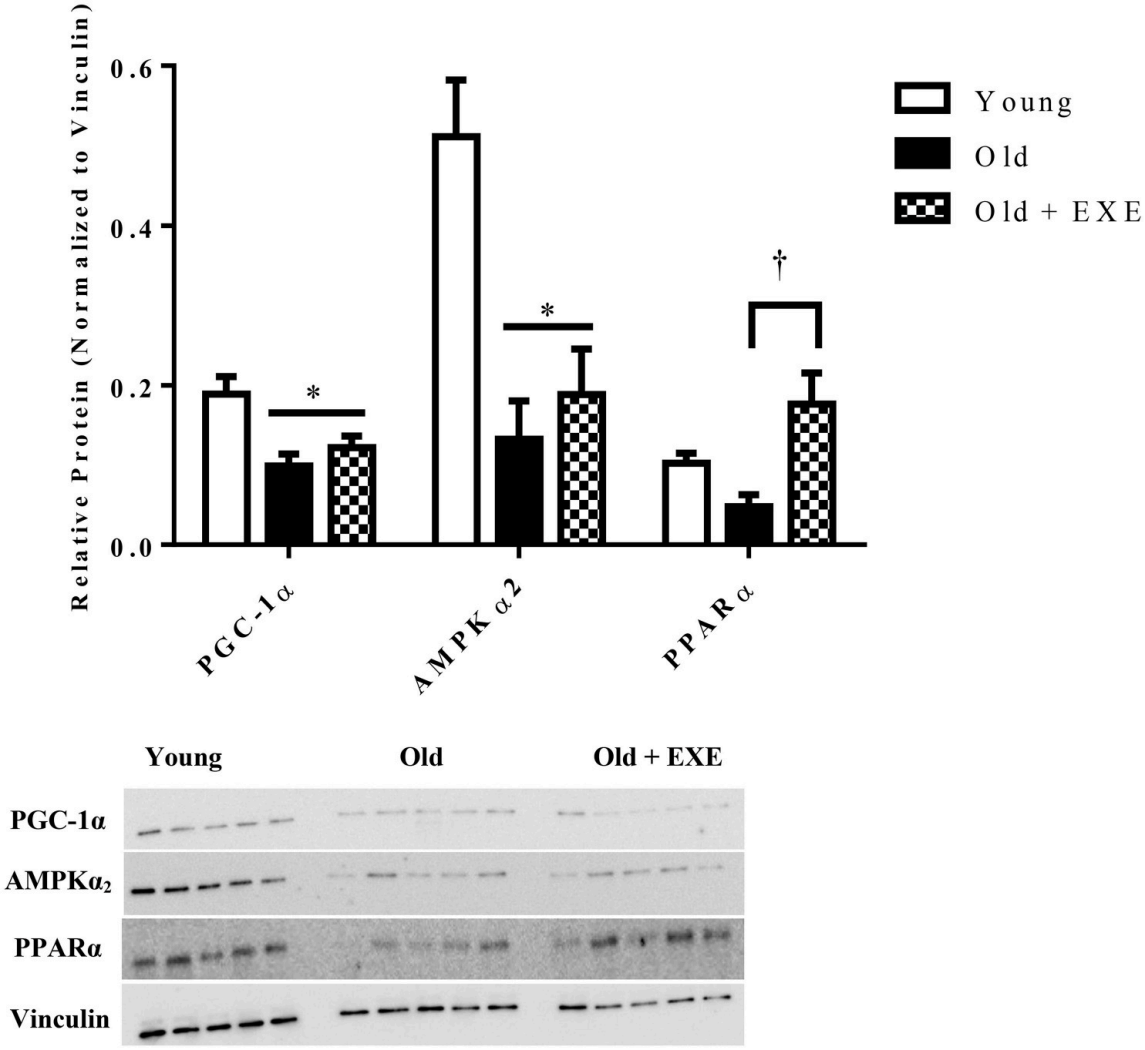
**Relative protein content in Young, Old, and Old + EXE groups (*n* = 5 per group)**. Values represent Means ± S.E.M. Old + EXE demonstrate increases in PGC-1α but decreased PPARα and AMPKα_2_ protein content compared to Young and Old, respectively. ^*^P < 0.05 vs. Young, ^†^*P* < 0.05 vs. Old + EXE.

In order to determine if the gene expression changes with age and exercise training were associated with altered protein content, we selected one protein from fatty acid oxidation (PPARα), AMPK signaling (AMPKα_2_), and mitochondrial biogenesis/function (PGC-1α) categories shown in Figure 2. Each of these genes had decreased mRNA expression and decreased protein content between Young and Old, although not significantly for PPARα (Figure 3). PGC-1α and AMPKα_2_ protein content was decreased in the Old + EXE compared to Young and not different than Old, but PPARα protein content was greater in the Old + EXE rat hearts compared to Old rat hearts.

### Citrate synthase activity

**Figure 4:**
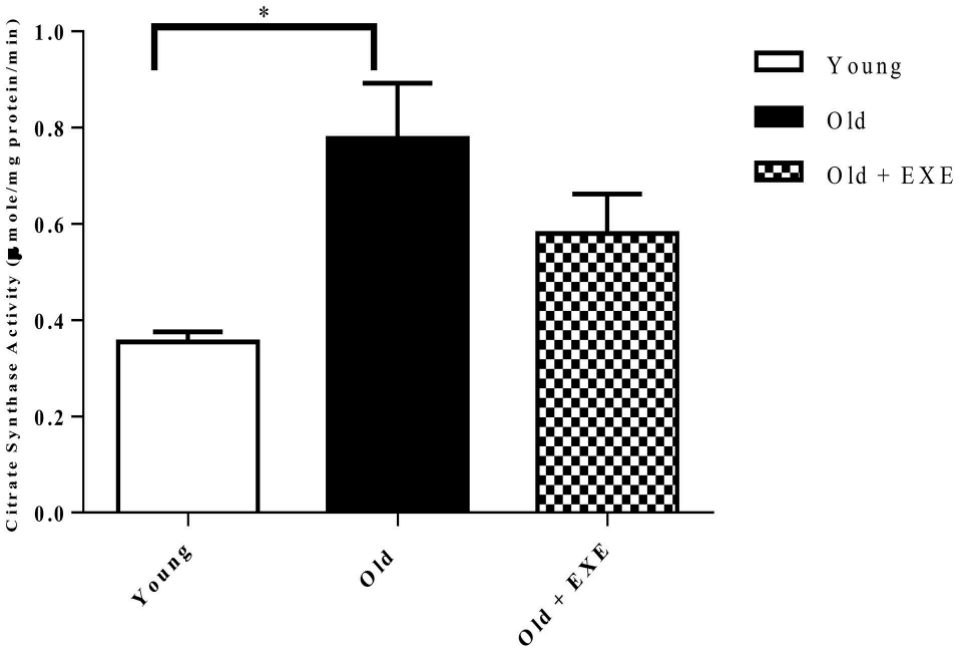
**Citrate synthase activity in left ventricular homogenates in young, old, and old exercise-trained hearts (*n* = 5 per group)**. Values indicate Means ± S.E.M. ^*^P < 0.05.

To determine mitochondrial function and volume we assayed citrate synthase activity in left ventricular homogenates. We found that Old rat hearts had increased citrate synthase activity compared to Young hearts, and the Old + EXE hearts demonstrated no differences in citrate synthase activity compared to Young or Old hearts (Figure 4).

## Discussion

To our knowledge this is the first study to determine the effects of age on the expression of a comprehensive group of genes related to cardiac substrate metabolism and mitochondrial function. The other primary aim of this study was to determine whether exercise training in aged rats could alter the age-related gene expression phenotype. We hypothesized that genes associated with fatty acid oxidation, AMPK signaling, and mitochondrial biogenesis/function would be decreased with age and that exercise training would mitigate these changes in aged rat hearts. We found that aging results in the decreased expression of many genes involved with energy metabolism and mitochondrial function but found that exercise training did not improve the downregulation of these genes. In fact, exercise training in aged rats resulted in the downregulation of 67 genes associated with energy metabolism and mitochondrial function compared to aged sedentary rat hearts. Genes associated with glucose metabolism were unaffected by age. The declines in metabolic gene expression were profound, as we observed decreased expression in 42 genes involved with fatty acid metabolism and mitochondrial function in aged hearts compared to young hearts. In aged sedentary hearts altered protein content of PGC-1α and AMPKα_2_ corresponded to declines in gene expression compared to young hearts. We observed that cardiac muscle citrate synthase activity; a common measure of mitochondrial volume in skeletal muscle (Larsen et al., 2012) was increased in the aged heart compared to the young heart, while exercise training in aged hearts showed no differences between young and old sedentary rat hearts. Overall, these results suggest a significant change in the expression of genes associated with cardiac metabolic pathways with age that is not improved with exercise training.

We determined that a large number of genes involved with fatty acid metabolism and mitochondrial energy metabolism/biogenesis were downregulated with age, consistent with previous reports suggesting age-related reductions in fatty acid oxidation (Abu-Erreish et al., 1977; McMillin et al., 1993; Kates et al., 2003), AMPK activity (Gonzalez et al., 2004; Turdi et al., 2010; Zhao et al., 2014), and mitochondrial function (Fannin et al., 1999; Wanagat et al., 2002; Kumaran et al., 2005; Bhashyam et al., 2007; Preston et al., 2008; Jian et al., 2011). Specifically, we found genes associated with FAO (i.e., CPT-2, HADHA), AMPK signaling (i.e., AMPKα_2_, CaMKK2, LKB1), and mitochondrial biogenesis (PGC-1α, PGC-1β) with age-related decrements in expression in the myocardium. Cardiac PGC-1α expression has previously been shown to decline with age (Preston et al., 2008; Turdi et al., 2010), although others have observed no age-related change in cardiac PGC-1α gene expression (LeMoine et al., 2006). Our results demonstrated that the genes associated with fatty acid oxidation that were downregulated with age were primarily PPARα regulated transcripts. As in previous studies, we found that expression of FAO genes downstream of PPARα were decreased (Iemitsu et al., 2002; LeMoine et al., 2006). However, our finding of a decline in PPARα regulated genes associated with FAO in aged hearts occurred despite no change in expression of the PPARα gene itself between young and old hearts. Although, this decline in genes associated with FAO is likely explained by reduced PPARα protein content in old hearts compared to young. This finding suggests that in the aged heart, changes in PPARα is likely to occur downstream of gene expression. Lastly, we demonstrated that the gene expression of AMPKα_2_ decreased with age, which is the major catalytic subunit in the heart and responsible for the phosphorylation of downstream proteins that confer AMPK's effect on energy metabolism in the heart (Dolinsky and Dyck, 2006). Also, we found decreased gene expression in the aging heart of two protein kinases (CaMKK2 & LKB1) that have been found to phosphorylate AMPK. These findings suggest that declining transcripts of AMPK and two upstream activators (CaMKK2 & LKB1), may play a role in the reduced AMPK activity that has been previously observed in aged hearts (Gonzalez et al., 2004; Turdi et al., 2010).

There is little information with regard to gene expression and protein content changes in exercise-trained aged rat hearts with regard to fatty acid oxidation, AMPK signaling, or mitochondrial function, but functional studies led us to hypothesize that exercise training would improve the age-related declines in metabolic gene expression. Surprisingly, we found that exercise training did not attenuate the age-related downregulation in the expression of genes involving fatty acid oxidation, AMPK signaling, and mitochondrial function. Specifically, in old exercise-trained hearts compared to old hearts we found a significant decline in the expression of the PPARα gene. PPARα regulated genes that were downregulated by exercise were Acyl CoA dehydrogenases (Acad), CD36, CPT1b, and CPT2. There were no differences in AMPK signaling genes (Figure 2B) between Old + EXE and Old rats; however, there was an upregulation of AMPKα_1_ in the exercise trained rats. AMPKα_1_ is ubiquitously expressed in cells and has lower levels of expression in the myocardium compared to AMPKα_2_ (Dolinsky and Dyck, 2006). We also found that compared to old rat hearts, exercise trained rat hearts demonstrated further downregulation of many genes involved with glucose transport (Glut4), Kreb's cycle and mitochondrial function (complex I and III in the electron transport chain). This downregulation of genes we observed in exercise trained aged hearts will require further work in order to better understand how exercise training induced downregulation of these genes affects cardiac function.

In order to determine if changes in gene expression with age were associated with altered protein content we measured the protein content of PGC-1α, PPARα, and AMPKα_2_. We found that the protein levels of PGC-1α and AMPKα_2_ were decreased in Old hearts compared to Young hearts and PPARα protein content trended toward a significant decline which was similar to the mRNA expression for these genes. In a previous report, mitochondrial oxygen consumption and expression of genes associated with mitogenesis and mitochondrial energy metabolism were both decreased, but mitochondrial number was increased and the mitochondrial marker, citrate synthase was not different in the senescent hearts compared to young (Preston et al., 2008). We observed a similar trend, of an increase in citrate synthase activity in Old hearts compared to Young hearts despite a decrease in gene expression of a large number of genes associated with mitochondrial biogenesis.

Western blot analysis determined that in the old-exercise trained rats PGC-1α and AMPKα_2_ protein content decreased compared to young hearts, similar to our findings of decreased gene expression of these two genes. However, we found that PGC-1α mRNA expression was reduced in Old + EXE hearts compared to Old hearts, yet the protein content of PGC-1α was similar. Also, we found that the PPARα mRNA expression was significantly reduced in Old + EXE hearts compared to Young and Old hearts, yet PPARα protein content was significantly greater in Old + EXE hearts compared to Old hearts and similar compared to Young hearts. These results are somewhat conflicted to a report demonstrating that exercise training attenuated the age-related decrement in PPARα mRNA expression and protein content in aged rat myocardium (Iemitsu et al., 2002). This study indicated that decreased mRNA expression did result in similar declines in protein content. However, functional changes do not always coincide with coordinate changes in gene expression or protein content (Burelle et al., 2004; Zhu et al., 2013; Lai et al., 2014). Changes in gene expression due to exercise training in aged hearts may or may not coincide with changes in that gene's protein product. Specifically, one study demonstrated that exercise training elicited adaptations that lead to an increase in exercise capacity along with augmented glucose and fatty acid oxidation in the myocardium despite no changes in the protein content of several proteins involved with glucose and fatty acid metabolism (Burelle et al., 2004). Our results demonstrated despite many genes involved with FAO in the exercise trained aged heart was reduced to aged sedentary hearts, PPARα protein content was greater in exercise trained aged hearts compared to aged sedentary hearts. Future studies looking at the protein content of major proteins involved with β-oxidation and fatty acid transport would enable us to see if exercise training in aged hearts tends to uncouple gene expression from protein content.

Citrate synthase is a common marker of mitochondrial volume used to indicate endurance exercise adaptations in skeletal muscle (Larsen et al., 2012). We examined citrate synthase activity in cardiac muscle and found, surprisingly, that aging resulted in an increase in citrate synthase activity and exercise training diminished this effect. Citrate synthase activity was not different in old-exercise trained hearts compared to young or old hearts. This is consistent with previous studies demonstrating that exercise training in rats did not elicit increases in cardiac citrate synthase activity (Oscai et al., 1971; Murakami et al., 1995; Zonderland et al., 1999; Siu et al., 2003; Rimbaud et al., 2009). We found that exercise training improved functional exercise capacity (Figure 4) despite either no changes in gene expression compared to Old hearts or in some cases, a further reduction in the expression of genes associated with energy metabolism and mitochondrial function in the heart. These data suggest that exercise training may impact myocardial energy metabolism and mitochondrial function downstream of gene expression. Also, exercise is known to induce adaptations in skeletal muscle (Hall et al., 1994; Bengtsson et al., 2001; Betik et al., 2008; Kang et al., 2013), which may have been responsible for the increased exercise capacity in our old exercise-trained rats.

## Limitations

One limitation to this study is that we did not determine whether exercise training in young rats leads to a similar downregulation of the expression of these cardiac genes that we found in the exercise trained aged hearts. Cardiac gene expression changes due to exercise training in young rats have been well-studied. These studies showed that mitochondrial or metabolic gene expression in the young rat heart to either increase (Hall et al., 1994; Rimbaud et al., 2009; Dobrzyn et al., 2013; Wadley et al., 2016) or not change (Murakami et al., 1995; Iemitsu et al., 2003; Alessio et al., 2014) with exercise training compared to young sedentary rats. Specifically, young hearts respond to exercise training by increasing the expression of genes associated with glucose transport (Hall et al., 1994; Rimbaud et al., 2009), fatty acid oxidation (Rimbaud et al., 2009; Dobrzyn et al., 2013), and mitochondrial biogenesis (i.e., PGC-1α and Cox4il; Rinaldi et al., 2013; Wadley et al., 2016). The results of our study (further decreases in gene expression with exercise training compared to sedentary aging) compared to these previous studies suggest that gene expression changes due to exercise training may be different in the hearts of aged exercise-trained rats compared to young hearts. Future works looking at post-translational modifications and protein activity in genes associated with fatty acid oxidation and mitochondrial function may elucidate molecular mechanisms involved in potential differential exercise training responses between young and old rat hearts.

Another limitation to this study is that our primary endpoint measure was the expression of genes associated with metabolic signaling pathways, substrate energy metabolism and mitochondrial function. Along with our data, previous reports have indicated that changes in tissue mRNA levels are not necessarily predictive of changes in protein levels (Hall et al., 1994; Zhu et al., 2013; Lai et al., 2014). Interestingly, other studies have reported that roughly two-thirds of the variance in protein content may be explained by mRNA concentration (Lu et al., 2007; Shankavaram et al., 2007). However, due to an increasing appreciation of miRNA interactions, post-translational modifications effecting protein activity, and the unpredictable protein response to stimuli (i.e., exercise training), mRNA is not always a good predictor of protein abundance (Burelle et al., 2004; Lu et al., 2007; Shankavaram et al., 2007). Future studies looking at expression changes in cardiac metabolic and mitochondrial genes, may need to include miRNA expression changes and post-translational protein modifications which will provide a more thorough understanding of metabolic and mitochondrial function in the aged and exercise trained rat heart.

## Conclusion

This study was the first to utilize a comprehensive approach in the study of age and exercise effects in aged hearts on substrate metabolism and mitochondrial function by an integration of gene expression, protein content, and protein activity. We found that cardiac aging results in the downregulation of a large number of genes associated with fatty acid metabolism, AMPK signaling and mitochondrial function, and exercise in aged-rats did not attenuate these changes and resulted in a further downregulation of expression in many metabolic and mitochondrial genes compared to aged sedentary rat hearts. We also found that gene expression changes may or may not coincide with protein expression. Taken together, our results demonstrated extensive age-related molecular changes in fatty acid metabolism, AMPK signaling and mitochondrial function. These molecular changes are far-reaching and cannot be described by changes in a single gene or a given gene's protein product.

## Author contributions

GB, GD, and JA designed the study. GB, SM, JS, and GD performed the experiments. GB, GD, JS, and SM analyzed the data. GB and GD drafted the manuscript and guarantors of the paper.

## Funding

This study was supported by National Institutes of Health grant AG030423 (JA).

### Conflict of interest statement

The authors declare that the research was conducted in the absence of any commercial or financial relationships that could be construed as a potential conflict of interest.
